# Comparing Match-Related Physical Performance Using Arbitrary and Individual Running Speed, Acceleration, and Deceleration Zones in Professional Football Players (Part 1)

**DOI:** 10.1519/JSC.0000000000005545

**Published:** 2026-05-29

**Authors:** Leo Antenucci, Christian Schmidt, Davide Malatesta

**Affiliations:** 1 Institute of Sport Sciences, Biomechanics & Integrative Physiology of Exercise Dynamics (BIPED) laboratory, University of Lausanne, Lausanne, Switzerland; and; 2 Performance Laboratory Football Club Lucerne, Lucerne, Switzerland

**Keywords:** GNSS tracking, intensity thresholds, match analysis, match load

## Abstract

Antenucci, L, Schmidt, C, and Malatesta, D. Comparing match-related physical performance using arbitrary and individual running speed, acceleration, and deceleration zones in professional football players (part 1). *J Strength Cond Res* 40(11): e1182–e1189, 2026—This study aimed to compare match-related physical performance and its match-to-match variability using arbitrary (ARB) and individualized (IND) intensity thresholds for running speed, accelerations (ACC), and decelerations (DEC) in a professional football team. Global navigation satellite system data from an entire Swiss first-division season (30 matches) were analyzed for 14 players. The match-related physical performance was assessed across 4 intensity zones for running speed, ACCs, and DECs: low-speed running, high-speed running (HSR), very high-speed running, sprinting and ACC_1/2/3/4_/DEC_1/2/3/4_. The distance covered and the number of ACCs/DECs in each zone were recorded through 3 thresholds for both ARB (speed: 14.4/19.8/25.2 km·h^−1^; ACCs/DECs: 1.0/2.0/3.0 m·s^−2^) and IND (speed: speed at the second lactate threshold, peak incremental speed, and anaerobic sprint reserve; ACCs/DECs: 25%/50%/75% of maximal ACCs/DECs). Within-player match-to-match variability coefficient of variation was also calculated. Significant differences were found between both methods across all zones (*p* ≤ 0.033). Running distances were significantly greater in IND than in ARB for all zones (4.5–110.7%) except HSR (−37.0%). Accelerations/DECs were significantly greater in IND for ACC_1_ (74.7%) and DEC_1_ (117.9%), but lower for ACC_2/3/4_/DEC_2/3/4_ (−16.0% to −96.3%). The number of ACC_4_/DEC_4_ was nearly negligible in IND. Coefficients of variation were lower for sprint distance, and greater for ACC_4_/DEC_4_ in IND than in ARB. The significant differences between ARB and IND running speed zones suggest that IND method should be used to assess match-related running performance accurately, while ARB method should be used for quantifying the number of ACCs/DECs.

## Abstract 2

Supplemental Digital Content is Available in the Text.

## Introduction

Increasing attention has been given to match-related physical performance and contextual factors to analyze team success. Football is a sport with high physiologic demands, involving intermittent efforts and numerous actions, such as sprints, jumps, and changes of direction, highlighting the crucial importance of developing both aerobic and anaerobic capacities (53). Players cover approximately 10–12 km per match, of which approximately 15% involves high-intensity running (HIR) (40). Although still a matter of debate (13,39), several studies have reported that total distance (TD), low-intensity running (LIR), HIR, very high-intensity running (VHIR), and sprint distance are greater in won matches than in those lost (11,21,41). In addition, the frequency of high-intensity accelerations (ACC) and decelerations (DEC) has also been found to be higher in winning performances (43). For these reasons, understanding and assessing the physiologic and metabolic demands of matches throughout different intensity zones and efforts are crucial.

According to the meta-analysis of Hader et al. (23), the most commonly used method to define intensity zones is based on arbitrary (ARB) and fixed absolute thresholds, including speed zones such as HIR (>14.4 km·h^−1^), VHIR (>19.8 km·h^−1^), and sprinting (>25.2 km·h^−1^). For ACC and DEC zones, thresholds are typically categorized as low, moderate, and high intensity based on absolute values (e.g., > or < ± 1.0/2.0/3.0 m·s^−2^, respectively) (23). However, several systematic reviews emphasize the importance of standardizing methods for both speed zones and ACC/DEC zones, because there is a lack of consensus among studies regarding the thresholds used to define the zones making the comparison of results among the studies difficult (7,23,24,49).

Some studies have proposed to individualize speed thresholds based on maximal sprint speed (MSS; HIR: ≥55% of MSS; VHIR: ≥70% of MSS) (5) or every 10% of MSS (45) or based on laboratory or field tests to measure physiologic capacities (26). No consensus exists regarding the methodology to adopt, and even individualized (IND) methods can be implemented in various ways, making it difficult to reliably compare results across studies and, therefore, it is crucial to clearly report the protocol used (16). Hunter et al. (26) proposed individualizing speed thresholds at the second lactate/ventilatory threshold (sLT_2_/sVT_2_), the velocity corresponding to maximal oxygen uptake (vV̇o_2_max), and the MSS. This approach better accounts for overall individual fitness levels and more accurately reflects each players' physical load, facilitating workload management by coaches and their physical conditioning staff, and reducing the risk of overtraining and injuries in football players (49). Indeed, based on previously reported values, noticeable divergences between ARB and IND methods can be inferred, with sLT_2_/sVT_2_ typically observed around 15 km·h^−1^ (2,4,22) and maximal aerobic speed (MAS) often ranging between 17 and 19 km·h^−1^ (4,31) in professional football players. Therefore, ARB thresholds may underestimate or overestimate each player's match load depending on their individual fitness level. Specifically, the distance covered in HIR zones (>14.4 km·h^−1^) may be artificially inflated with the ARB approach, whereas the distance covered in VHIR zones (>19.8 km·h^−1^) may be underestimated compared with IND thresholds.

Match-to-match variability in running performance may arise from both methodological and contextual sources, because factors such as formation, ball possession, scoreline, and match congestion can influence match-running demands (55). Part of this variability likely reflects the influence of individual pacing strategies, because players regulate their physical exertion to remain within their physiologic limits (14), which is pivotal in reducing the inherent match-to-match variability in HIR distance (22). High-intensity running efforts (≥19.8 and ≥25.2 km·h^−1^) showed higher variabilities (approximatively 17–20% and 30–38%, respectively) than more stable indicators such as TD (<6%) (22,37) or lower-intensity and IND thresholds (≥80%: 11.8%; >100% MAS: 17.0%) (15). Moreover, match-to-match variability in HIR distance seemed to be lower when using the IND compared with the ARB thresholds, suggesting the IND approach may provide a more stable indicator of running performance during matches (15).

Although less investigated in the literature, Abbott et al. (1) also highlighted differences between the ARB and IND thresholds for ACC in young football players. These authors recommended defining IND ACC zones based on a percentage of each player's maximal ACC capacity (ACC_max_) obtained from a maximal sprint. This approach avoids the overestimation of ACC distances observed with the ARB method in more explosive players. Only a few studies have analyzed the match-to-match variabilities of ACCs/DECs, reporting lower variabilities for the total number of ACCs (ACC_TOT_) (8,37) and DECs (DEC_TOT_) (8) than high-intensity running efforts. However, to the best of our knowledge, no study has yet investigated the variability of ACCs and DECs at different intensities.

This study aimed to compare match-related physical performance and its match-to-match variabilities using ARB and IND thresholds for running speed and ACCs/DECs in a professional football team for an entire sporting season. We hypothesize that (a) HIR distance would be greater and VHIR distance lower when using ARB than when using IND thresholds; (b) the number of high-intensity ACCs/DECs would be higher in the ARB than in the IND method; (c) match-to-match variability would increase with intensity across running speeds, ACCs, and DECs; and (d) the IND method would result in lower variabilities for high-intensity efforts than the ARB method.

## Methods

### Experimental Approach to the Problem

This study used a single-cohort, observational, repeated-measures design aimed at comparing match-related physical performance between ARB and IND thresholds across an entire competitive season. Players' physical performance during matches was assessed using a 10 Hz global navigation satellite system (GNSS; APEX Pro Series Units, STATSports, Northern Ireland), which has been previously validated (9,30). The mean number of satellites connected during matches was 17.7 ± 1.3 (range: 14–21), in line with the literature (5,10). The GNSS units were placed in the pouch of a vest worn at the upper back under the team jersey. After each match, data were downloaded through Sonra software (Sonra 4.0, STATSports), then converted and exported to an Excel file for analysis.

### Subjects

The Championship and Swiss Cup matches of a Swiss Super League professional team comprising 33 field players who participated in at least 1 official match during the 2022–2023 season were analyzed. Throughout the season, the squad participated in 1 to 2 matches per week, in addition to 4 to 5 specific and conditioning training sessions. Of the 33 players, only 14 met the inclusion criteria across 27 Super League and 3 Swiss Cup matches, mainly because of limited playing time of youth players, injuries, or transfers. The inclusion criteria were as follows: (a) starting players who played at least 2 entire matches (i.e., 90 minutes) to ensure representative performance data, (b) no injury sustained by the player during the match, and (c) matches lasting 90 minutes with 11 players on each side (no red cards during the match) to avoid the potential impact of numerical inferiority on physical performance. Substitute players were not included because, although some studies report conflicting results, substitutes generally display higher physical output than starting players during equivalent periods of play (25). Additional time at the end of each half-time and extra-time during cup matches was not considered to keep homogeneity between matches. The team played with several formations during the entire season: 4-4-2 (*n* = 1), diamond 4-4-2 (*n* = 13), 4-2-3-1 (*n* = 12), and 4-1-4-1 (*n* = 4). However, tactical formations were not included as covariates in the analysis, because the primary aim of the study was to compare ARB and IND methods irrespective of match context. No specific intervention was required for this study, which was performed according to the Declaration of Helsinki and for which each player signed a written consent to be included in this study. The sample size was determined a priori using a 2-tailed paired *t* test, assuming an effect size (0.86), derived from a previous study that compared VHSR distances obtained with ARB and IND methods similar to those used in our study (42). Statistical power was set at 0.80, with a significance level of 0.05 (18,58). This analysis indicated that a minimum of 10 subjects would be sufficient; however, 14 were recruited to increase statistical power.

### Procedures

#### Match-Play Physical Performance Assessment

The distance covered at 4 speed intensities was assessed: low-speed running (LSR), high-speed running (HSR), very high-speed running (VHSR), and sprint distance (for more details, see the paragraph “Intensity zones”). Furthermore, HIR (HSR + VHSR + sprint distance) and VHIR (VHSR + sprint distance) were also evaluated. The number of ACCs/DECs performed during the match was also divided into 4 intensity zones: very low-intensity (ACC_1_/DEC_1_), low-intensity (ACC_2_/DEC_2_), moderate-intensity (ACC_3_/DEC_3_), and high-intensity (ACC_4_/DEC_4_; for more details, see the paragraph “Intensity zones”), and the number of >ACC_3_ (ACC_3_ + ACC_4_) and >DEC_3_ (DEC_3_ + DEC_4_) were also calculated. The number of ACCs/DECs in each ACC and DEC zone was used because this metric seems to be more appropriate than the distance covered in these intensity zones for assessing match-related physical performance in football (44).

Data for each intensity zone of running speed, ACCs, and DECs were extracted using both IND and ARB methods, and within-player match-to-match variability was calculated for each method (for more details, see the paragraph “Within-player match-to-match variability”). Because IND zones were updated in the second part of the season, variability was calculated separately for the first and second parts of the season and then averaged for each player and for the team. Of the 14 players included in the comparison between ARB and IND methods, only 13 were retained in the variability analysis, because players were required to have played at least 2 matches within the same half of the season to calculate variability.

#### Intensity Zones

The speed, ACC, and DEC ARB zones were set according to thresholds defined in the literature (23) and are presented in Table 1. The IND speed zones were defined on the basis of physiologic thresholds [i.e., speed associated with the first lactate thresholds (sLT_1_), sLT_2_, and the peak incremental speed (PIS)] that reflect individual metabolic and performance characteristics, following the approach provided by Hunter et al. (26) (Table 1). The IND ACC and DEC zones were determined relative to each player's ACC_max_ and maximal deceleration (DEC_max_) capacities, based on thresholds used in previous studies (1,3,14) (Table 1). The duration of each ACC/DEC must exceed 0.5 seconds for them to be counted in each intensity zone.

**Table 1:** Arbitrary (ARB) and mean individual (IND) intensity zones for speed, acceleration (ACC), and decelerations (DEC).*

		Zone 1	Zone 2	Zone 3	Zone 4
ARB					
Speed	km·h^−1^	<14.4	14.4–19.8	19.8–25.2	>25.2
ACC	m·s^−2^	0.5–1.0	1.0–2.0	2.0–3.0	>3.0
DEC	−m·s^−2^	0.5–1.0	1.0–2.0	2.0–3.0	>3.0
IND					
Speed	Thresholds	< sLT_2_	sLT_2_–PIS	PIS–30% ASR	30% ASR–MSS
	km·h^−1^	<15.0	15.0–18.4	18.4–23.1	23.1–34.1
ACC	Thresholds	<25% ACC_max_	25–50% ACC_max_	50–75% ACC_max_	>75% ACC_max_
	m·s^−2^	0.50–1.55	1.55–3.10	3.10–4.65	4.65–6.20
DEC	Thresholds	<25% DEC_max_	25–50% DEC_max_	50–75% DEC_max_	>75% DEC_max_
	−m·s^−2^	0.50–2.06	2.06–4.12	4.12–6.19	6.19–8.25

* Zone 1: low-speed running (LSR), very low-intensity acceleration/deceleration (ACC_1_/DEC_1_); Zone 2: high-speed running (HSR), low-intensity acceleration/deceleration (ACC_2_/DEC_2_); Zone 3: very high-speed running (VHSR), moderate-intensity acceleration/deceleration (ACC_3_/DEC_3_); Zone 4: sprint distance, high-intensity acceleration/deceleration (ACC_4_/DEC_4_). ASR = anaerobic speed reserve; MSS = maximal sprint speed; PIS = peak incremental speed; sLT_2_ = speed associated with the second lactate threshold.

To define the IND speed zones for each player, a laboratory discontinuous incremental treadmill test to exhaustion was conducted during the preseason (June–July) and the mid-season (January) to individually determine the sLT_1_, sLT_2_, and PIS. The second incremental test was used to update the IND thresholds for the second part of the season. Although aerobic and anaerobic capacities may evolve during the competitive period, previous studies have shown that most aerobic adaptations occur during the preseason, with only minor or specific changes thereafter (28,33). Therefore, conducting 2 assessments (pre- and mid-season) was considered sufficient to capture meaningful changes in players' aerobic fitness. The treadmill was set at a 1.0% incline with an initial speed of 7.2 km·h^−1^, and an increase of 1.8 km·h^−1^ every 3 minutes and 30 seconds (with 30 seconds of rest for lactate sampling from the earlobe analyzed with Biosen C-Line, EKF Diagnostic, Barleben, Germany). Peak incremental speed was defined as the running speed of the last fully completed step (S_last, completed, step_) plus the fraction of time spent in the following uncompleted step (α) multiplied by the running speed increment (Δ*s* = 0.01 km·h^−1^) (29): PIS = S_last, completed, step_ + αΔs. sLT_1_ corresponds to the speed at which lactate accumulation begins slightly and sLT_2_ is set at the second break in the speed/lactate relationship where a more significant increase in lactate accumulation occurs (12).

Owing to logistical constraints and to minimize injury risks, because match values may exceed those recorded during a 40-m sprint test (32), the club did not conduct sprint tests with determination of MSS, ACC_max_, and DEC_max_. These maximal values were extracted from both friendly and official matches, a method that allows assessing players' physical performance in real competition conditions. Laboratory tests were conducted twice (in June and January) to monitor the evolution of players' capacities. The MSS, ACC_max_, and DEC_max_ for each player were extracted from each half of every match up to the last Super League match of the first and second half of the season. To define the MSS and ACC_max_/DEC_max_ of each player to establish the IND thresholds, the boxplot method was used, using the inter quartile range (IQR = third quartile − first quartile) to eliminate potential outliers (>third quartile +1.5 × IQR) (19). After the MSS is determined, the anaerobic speed reserve (ASR) can also be calculated (i.e., ASR = MSS-PIS) (34).

#### Within-Player Match-To-Match Variability

The within-player match-to-match coefficient of variation [CV: (*SD*/mean × 100)] was calculated for each player using both IND and ARB methods. When available, the 2 seasonal CVs (first and second halves) were averaged to obtain a single CV per player.

### Statistical Analysis

All the statistical analyses were conducted through Jamovi software (version 2.3; Sydney, Australia (54)). The results are expressed as mean ± *SD*. For each intensity zone, a linear mixed model was used to compare running distances, ACCs and DECs, between both methods (ARB and IND). The dependent variables were the distance covered in each speed-intensity zone, the number of ACCs or DECs performed in each respective zone were the dependent variables (i.e., 1 analysis per intensity zone). The fixed effect was the “method,” with the player as the clustering variable. The “method|player” was selected as the random coefficient. A linear mixed model was used with 2 fixed effects to compare the results between both methods (ARB and IND) and between intensity zones in each method.

The 2 fixed effects were “method” and “intensity zone,” with the player as the cluster variable, while CV was the dependent variable. The “intercept|player” was selected as the random coefficient. For HIR, VHIR, >ACC_3_, and DEC_3_, a linear mixed model was used to separately compare the CVs between both methods (ARB and IND). The fixed effect was the “method,” with the player as the clustering variable. The “intercept|player” was selected as the random coefficient.

For all linear mixed models used, post hoc comparisons were performed with the Bonferroni correction to take the repeated pairwise comparisons into account. A significant difference was set at *p* ≤ 0.05.

## Results

### Subjects

The analysis identified 14 players (age: 23.9 ± 4.5 years (range: 18 - 32 years); height: 181.8 ± 4.7 cm; body mass: 76.6 ± 5.9 kg; body mass index: 23.2 ± 1.2 kg/m^2^) who completed at least 2 full matches, totaling 178 observations for each method. The team's mean IND zones (average values over the season) obtained from the physical tests and maximal values are given in Table 1 (sLT_1_: 10.7 ± 0.7 km·h^−1^; sLT_2_: 15.0 ± 0.8 km·h^−1^; PIS: 18.4 ± 0.7 km·h^−1^; maximal heart rate (HR_max_): 193.8 ± 6.9 bpm; maximal blood lactate concentration ([La]_max_): 11.3 ± 1.8 Mmol; MSS: 34.1 ± 1.6 km·h^−1^; ACC_max_: 6.2 ± 0.3 m·s^−2^; DEC_max_: −8.3 ± 0.6 m·s^−2^).

### Differences Between the Arbitrary and the Individual Methods

The fixed effects of distances covered and the number of ACCs and DECs in each intensity zone for both the ARB and IND methods are presented in Table 2. A significant fixed effect between methods was found for all speed, ACC, and DEC intensity zones (*p* ≤ 0.004). The LSR, VHSR, VHIR, and sprint distance covered were significantly greater with the IND method than with the ARB method (+4.5%, +20.3%, +37.0% and +100.7%, respectively; *p* ≤ 0.004), whereas the HSR and HIR distance covered were significantly lower with the IND method than with the ARB method (−37.3% and −14.3%, respectively; *p* ≤ 0.001). The number of ACC_1_/DEC_1_ performed per match was significantly greater with the IND method than with the ARB method (+74.7% and +117.9%, respectively; *p* < 0.001), whereas the opposite result was found for all other intensities (ACC_2_: −16.0%; ACC_3_: −55.9%; >ACC_3_: −68.0%; ACC_4_: −90.6%; DEC_2_: −42.1%; DEC_3_: −77.0%; >DEC_3_: −84.2%; DEC_4_: −96.3%; *p* < 0.001).

**Table 2:** Mean ±*SD* distance covered, number of accelerations (ACC), and decelerations (DEC) performed in each intensity zone.*

Metric	ARB	IND†	IND/ARB (%)	Method effect (*p*)
LSR (m)	7,544 (389)	7,880 (565)	4.45%	0.004
HSR (m)	1,624 (357)	1,018 (250)	−37.32%	<0.001
HIR (m)	2,354 (518)	2017 (429)	−14.32%	0.001
VHSR (m)	577 (164)	694 (179)	20.28%	<0.001
VHIR (m)	729 (210)	999 (244)	37.04%	<0.001
Sprint distance (m)	152 (72)	305 (101)	100.66%	<0.001
ACC_1_ (n)	221 (23)	386 (39)	74.66%	<0.001
ACC_2_ (n)	257 (26)	216 (27)	−15.95%	<0.001
ACC_3_ (n)	119 (18)	52.5 (14.8)	−55.88%	<0.001
>ACC_3_ (n)	183 (29)	58.6 (18.4)	−67.98%	<0.001
ACC_4_ (n)	64.1 (15.4)	6.04 (5.16)	−90.58%	<0.001
DEC_1_ (n)	218 (29)	475 (50)	117.89%	<0.001
DEC_2_ (n)	252 (29)	146 (28)	−42.06%	<0.001
DEC_3_ (n)	102 (15)	25.5 (11.9)	−75.00%	<0.001
>DEC_3_ (n)	180 (26)	28.4 (15.2)	−84.22%	<0.001
DEC_4_ (n)	77.3 (18.2)	2.83 (4.94)	−96.34%	<0.001

* LSR = low-speed running; HSR = high-speed running; HIR = high-intensity running; VHSR = very high-speed running; VHIR = very high-intensity running; ACC_1_/DEC_1_ = very low-intensity acceleration/deceleration; ACC_2_/DEC_2_ = low-intensity acceleration; ACC_3_/DEC_3_ = moderate-intensity acceleration/deceleration; >ACC_3_/>DEC_3_ = ACC_3_ + ACC_4_ and DEC_3_ + DEC_4_, respectively; ACC_4_/DEC_4_ = high-intensity acceleration/deceleration; ARB = arbitrary; IND = individual.

† Significant fixed effect between methods (*p* ≤ 0.05).

The fixed effects of CV in each intensity zone for both ARB and IND methods are presented in Table 3. A fixed effect between methods was significantly found for sprint distance, ACC_4_, and DEC_4_ (*p* < 0.001 for all) and was not significant for LSR, HSR, VHSR, ACC_1/2/3_, and DEC_1/2/3_ (*p* ≥ 0.085). The CV of sprint distance was significantly lower with the IND method than with the ARB method. The CV of ACC_4_ and DEC_4_ was greater with the IND method than with the ARB method. Differences between intensities for separately both ARB and IND methods are presented in Table 3. The CV of LSR was significantly lower than that of HSR, VHSR, and sprint distance (*p* ≤ 0.01), and the CV of HSR and VHSR was significantly lower than that of sprint distance (*p* ≤ 0.002) for both ARB and IND methods. Differences of CVs across all ACC and DEC intensities were not significant with the ARB method (*p* ≥ 0.176). The CV of ACC_1_ was significantly lower than that of ACC_3_ and ACC_4_ in IND (*p* ≤ 0.021). The CV of ACC_2_ was significantly lower than that of ACC_4_ in IND (*p* < 0.001). The CV of ACC_3_ was significantly lower than that of ACC_4_ in IND (*p* ≤ 0.021). The CV of DEC_1_, DEC_2_, and DEC_3_ was significantly lower than that of DEC_4_ in IND (*p* < 0.001).

**Table 3:** Within-player coefficients of variation (CV%) and 95% confidence interval for running distance, number of accelerations (ACC), and decelerations (DEC) performed in each intensity zone.

Metric	ARB	IND	Method effect (*p*)
LSR (%)	3.54 [2.64–4.45]^2,3,4^	3.51 [2.56–4.45]^2,3,4^	0.988
HSR (%)	11.4 [9.2–13.5]^1,4^	12.5 [9.8–15.2]^1,4^	0.643
VHSR (%)	15.0 [12.3–17.8]^1,4^	12.2 [9.9–14.4]^1,4^	0.238
Sprint distance (%)	30.6 [21.8–39.4]^1,2,3^	21.6 [14.3–28.9]^1,2,3^*	<0.001
ACC_1_ (%)	7.21 [5.77–8.66]	5.83 [4.51–7.15]^A3,A4^	0.573
ACC_2_ (%)	7.43 [6.74–8.11]	8.49 [6.46–10.50]^A4^	0.665
ACC_3_ (%)	11.0 [9.1–12.8]	13.2 [10.0–16.4]^A1,A4^	0.368
ACC_4_ (%)	12.6 [10.4–14.9]	43.8 [34.1–53.5]^A1,A2,A3^*	<0.001
DEC_1_ (%)	7.37 [5.71–9.03]	4.82 [3.79–5.86]^D4^	0.706
DEC_2_ (%)	5.55 [4.12–6.98]	8.87 [7.63–10.10]^D4^	0.623
DEC_3_ (%)	10.9 [9.4–12.3]	22.6 [18.4–26.7]^D4^	0.085
DEC_4_ (%)	11.6 [8.4–14.8]	89.1 [60.3–118.0]^D1,D2,D3^*	<0.001

* Significant fixed effect between methods; 1/2/3/4 = post hoc significant difference with LSR/HSR/VHSR/sprint for the same method; A1/A2/A3/A4 = post hoc significant difference with ACC_1_/ACC_2_/ACC_3_/ACC_4_ for the same method; D1/D2/D3/AD = post hoc significant difference with DEC_1_/DEC_2_/DEC_3_/DEC_4_ for the same method (*p* ≤ 0.05).

## Discussion

The purpose of this study was to compare match-related physical performance and match-to-match variability between the ARB and IND thresholds. Our results revealed notable differences between these 2 methods, confirming our hypothesis. Specifically, the distances covered in HSR and HIR were greater with the ARB method, whereas LSR, VHSR, VHIR, and sprint distances were higher with the IND method. Furthermore, the number of ACCs and DECs was greater with ARB thresholds across all intensity zones except very low intensity (i.e., ACC_1_/(DEC_1_): (−) 0.5 − (−) 1.0 m·s^−2^). Match-to-match variability increased with intensity for running speed, ACCs, and DECs, confirming our hypothesis. However, the IND method showed significantly lower CV only for sprint distance, while ACC_4_/DEC_4_ exhibited higher variability with this method, partially contradicting our initial expectations.

When comparing thresholds between methods, LSR, HSR, VHSR, and Sprint were delimited at 14.4, 19.8, and 25.2 km·h^−1^ in ARB, and in average at 15.0, 18.4, and 23.1 km·h^−1^ in IND. Compared with ARB, the IND threshold for the LSR–HSR transition was slightly higher (15.0 vs. 14.4 km·h^−1^), while the lower limits for VHSR (18.4 vs. 19.8 km·h^−1^) and sprinting (23.1 vs. 25.2 km·h^−1^) were lower. These differences led to greater distances covered in LSR (+4.4%), VHSR (+20.6%), and sprinting (+101.3%) with the IND method. Although IND zones were narrower for VHSR (18.4–23.1 km·h^−1^ vs. 19.8–25.2 km·h^−1^), the lower absolute intensities made it easier to accumulate distance at moderate speeds. However, this principle does not apply to HSR, where ARB uses both a lower lower-limit (14.4 vs. 15.0 km·h^−1^) and a higher upper-limit (19.8 vs. 18.4 km·h^−1^), resulting in a smaller HSR distance (−37.3%) with IND. For example, a player with a PIS of 21 km·h^−1^ and MSS of 35 km·h^−1^ (30% ASR) would reach a sprint threshold of 25.2 km·h^−1^ in both methods, but his VHSR range would shift downward in IND (21.0–25.2 km·h^−1^) compared with ARB (19.8–25.2 km·h^−1^), leading to a different distribution of efforts across intensity zones (Table 1). This example highlights the importance of individualizing intensity thresholds to accurately and fairly determine each player's relative physical load, as already shown for properly prescribing endurance training using speed at lactate or ventilatory thresholds (27,35,47,57) and ASR (46,56). Silva et al. (50) reported that the ARB threshold for sprinting (≥25.2 km·h^−1^) is obsolete because it represents a relative intensity ranging from 71 to 91%, depending on each player's MSS capacity, leading to inaccurate individual match load assessment. The validity of the acceleration–speed profile during matches has already been demonstrated (36), so the MSS during matches can certainly be reliable. However, it would still have been interesting to analyze the differences in the delimitation of VHSR and sprint using MSS from matches vs. MSS from an isolated sprint, as deriving MSS from match data may slightly underestimate true maximal values for players who did not reach top speed during competition, which represents a potential limitation of this study. However, evidence indicates that MSS values derived from match data are comparable with, or even exceed, those obtained in standardized 40-m sprint tests (32), supporting the validity and safety of this ecological approach.

Moreover, standardizing techniques for determining lactate thresholds following the principles presented by Jamnick et al. (27) is crucial but not always respected. The use of fixed values (2 mmol·L^−1^ and 4 mmol·L^−1^, for sLT_1_ and sLT_2_, respectively) should no longer be considered because it may distort IND zones and misrepresent players' actual loads. Determining physiologic markers such as PIS, MSS, and ASR is, therefore, essential to tailor training prescriptions and manage overall load according to each player's aerobic and anaerobic profile (46). However, the lack of consensus on threshold categorization and the diversity of IND methods used across studies (16,23,49) still complicate comparisons and underline the need for standardized, validated approaches. Overall, these findings emphasize the importance of IND zones to consider individual physical capacity differences and better monitor players' load throughout the season. Therefore, when laboratory assessments are not feasible because of logistical or financial constraints, field-based tests to determine MAS and MSS remain a valid and practical alternative to IND thresholds (26). A limitation is that the physiologic tests to determine IND thresholds were conducted during the preseason period, when players may still improve their fitness (28,33). Therefore, conducting the tests at the end of the preseason, just before the start of the season, would likely provide a more accurate assessment of players' IND thresholds. However, owing to logistical and scheduling constraints (e.g., injuries, transfers), preseason remains the most practical window for assessments. Despite this, IND thresholds still offer a more accurate representation of players' capacities compared with ARB thresholds. The approach used in this study, based on individual physiologic values (i.e., sLT_2_, PIS, and 30% ASR), provides a strong solution, having been suggested (26) and validated (17,27,46) in the literature.

Studies using IND thresholds for ACCs are limited, complicating the understanding and interpretation of this variable. To the best of our knowledge, the only study comparing the ARB and IND methods reported greater distances covered in ACC_2/3/4_ with the ARB than the IND method (1), which corroborates our results (Table 2). Systematic reviews have also highlighted the inconsistency in ACC zones definitions and recommended IND thresholds as a percentage of ACC_max_ (24,48). However, ACC_max_ decreases linearly as initial running speed increases (52), meaning that achieving ACC >3 m·s^−2^ above 15 km·h^−1^ becomes nearly impossible for many players. As a result, ACCs performed at high speeds are often classified as low- or moderate-intensity despite their substantial physiologic cost, leading to a likely underestimation of the real load. Although the number of ACCs in ARB and IND showed similarities, the boundaries shifted between methods (Table 1). For example, the lower limit of ACC_4_ in ARB (>3 m·s^−2^) corresponded to ACC_3_ in IND (3.07 m·s^−2^). Moreover, the very low number of ACC_4_ performed in IND (*n* = 6.0) questions the practical relevance of individualizing ACC thresholds using this approach. Therefore, despite potential biases associated with the ARB method, individualization based solely on ACC_max_ does not seem to improve the interpretation of players' mechanical load. Some studies have proposed individualizing ACC thresholds according to the running speed at the start of each effort, which may more accurately reflect the mechanical demands (20,51). However, these approaches require extensive processing of raw GNSS data and are not currently implemented in commercial systems, making them impractical for technical staff in applied settings. Until GNSS software integrates speed-dependent IND ACC thresholds, the ARB method remains, in our opinion, the most pragmatic option.

Our study is the first to propose an IND method based solely on DEC_max_, making comparisons with the literature and drawing clear conclusions difficult. However, corroborating our hypothesis, the DECs results showed similar patterns to those for ACCs. With the IND method, the number of DEC_1_ was markedly higher (+118.4%), whereas DEC_2/3/4_ were substantially lower (−42.1%, −74.9%, and −96.3%, respectively; Table 2). Unlike ACCs, where IND and ARB thresholds appeared shifted, the IND DEC zones were nearly twice as wide as those in ARB because of the higher DEC_max_ value relative to ACC_max_. For example, IND DEC_1_ (0.50–2.04 m·s^−2^) closely matched ARB DEC_1_ + DEC_2_ (0.5–2.0 m·s^−2^), and IND DEC_2_ extended up to 4.08 m·s^−2^ compared with 2–3 m·s^−2^ in ARB (Table 1). Similarly to ACC_4_, IND DEC_4_ counts were almost absent (*n* = 2.8 vs. 77.3 in ARB), and IND DEC_3_ counts were much lower (*n* = 25.6 vs. 102.0, respectively; Table 2). Together, these results suggest (a) limited relevance of IND-based DEC zones with the current approach, and (b) that setting a higher DEC_4_ threshold (e.g., < −4 m·s^−2^) is unlikely to provide more meaningful information despite the higher DEC_max_. Similarly to ACCs, although 1 study has already proposed individualizing DEC thresholds based on initial running speed (51), this approach faces the same practical limitations as previously discussed. Therefore, integrating speed-dependent DEC thresholds into GNSS software, consistent with the developments needed for ACCs, would allow future studies to investigate IND DECs across various intensities and improve the accuracy of mechanical and physiologic load quantification.

Previous studies have shown that match-to-match variability increases with running intensity, reporting CVs ranging from 17 to 20% for VHSR and from 30 to 38% for sprinting (15,22,37). Consistent with these findings, our results also showed a progressive increase in variability with intensity, with CVs of 3.5, 11.4, 15.0, and 30.6% for LSR, HSR, VHSR, and sprint distance, respectively, using the ARB method (Table 3). Carling et al. (15) also reported lower CV in HIR when using IND thresholds based on MAS (17.0%) rather than ARB (19.8%). Our results partly support this observation. Indeed, sprint distance showed a clear and significant lower variability with IND than with ARB, and VHSR also displayed lower CV values with IND, although this difference was not statistically significant (Table 3). More importantly, the IND method based on physiologic markers (sLT_2_, PIS, and 30% ASR) yielded more stable CVs across intensities. HSR and VHSR showed almost identical CVs with IND (12.5 and 12.2%), whereas ARB displayed a clear, although not statistically significant, upward progression (11.4–15.0%). Finally, sprint variability was much lower with the IND method than with ARB, resulting in both lower and narrower overall CV values with IND (3.51–21.6%) than with ARB (3.54–30.6%), indicating that IND physiologically derived thresholds provide a more stable and reliable assessment of running performance across matches.

Regarding ACCs and DECs, variability followed a similar pattern, increasing with intensity with both methods (Table 3). To the best of our knowledge, this is the first study to assess variability across different ACC/DEC intensities. Previous studies focused only on ACC_TOT_/DEC_TOT_ and reported within-player CVs ranging from 2.6 to 9.7% (8,37), comparable values to the present study (5.6–12.6% across all ACC/DEC intensities). Importantly, unlike running speed zones, ACC/DEC zones, and particularly ACC_4_/DEC_4_, consistently showed lower CVs with the ARB method than with IND, indicating that ARB provides a more stable and reliable classification for these metrics. Finally, with ARB, higher-intensity zones (ACC_3_/DEC_3_, >ACC_3_/>DEC_3_ and ACC_4_/DEC_4_) also seemed less variable than VHSR and sprinting when using IND (10.9–12.6% vs. 12.2–30.6% for ACCs/DECs and running speed, respectively), suggesting that ACCs and DECs may represent more stable match-to-match indicators for individual players.

Given the practical limitations of individualizing ACCs/DECs with raw GNSS data, we initially used a simplified IND approach to evaluate these metrics, which was feasible for technical staff in applied settings. Although data-driven or clustering-based statistical methods (e.g., k-means or spectral clustering) could potentially refine the definition of ARB thresholds (38), these approaches have not yet been clearly validated in football and do not address the need for physiologic individualization. Future studies could explore their integration once their practical and theoretical relevance has been established. However, our findings showed that the traditional ARB method was more stable and logical than the IND method only based on ACC_max_/DEC_max_. In contrast, the IND thresholds for running speed seemed more suitable, providing a better representation of individual physical capacity. These results suggest that combining IND thresholds for speed zones with ARB thresholds for ACCs and DECs offers the most practical and accurate strategy for analyzing match-related physical performance in professional football players, as long as GNSS software has not yet implemented the IND method accounting for initial running speed. Based on these novel findings, IND speed zones and the ACC and DEC ARB zones have been used to investigate the relationship between match-related physical performance decline and physical fitness parameters in professional football players for an entire season (6).

**Practical Applications**

This study demonstrated that the choice of method (ARB vs. IND) considerably influences the assessment of match-related physical performance and its variability. IND thresholds for running speed provided more individualized and lower variability, resulting in more relevant evaluations of player load. Despite the challenges of comparing studies that use different thresholds, coaching staffs should prioritize the IND method to better monitor individual player loads. However, ARB thresholds seemed more appropriate for ACCs and DECs, offering greater consistency in variability and practicality. These findings support a mixed-method approach for coaching staffs: IND thresholds for running speeds and ARB thresholds for ACC and DEC zones. In contrast, using standardized ARB thresholds for all metrics (running speed, ACCs and DECs) could be useful for comparisons between teams or competitions at broader levels (e.g., federations, performance analysis platforms, or studies involving entire leagues). Finally, future developments in GPS software should include systems that account for each player's acceleration–velocity profile and generate corresponding IND zones.
